# Gene Therapy: An Historical Overview for Familial Hearing Loss

**DOI:** 10.3390/ijms26041469

**Published:** 2025-02-10

**Authors:** O’neil W. Guthrie

**Affiliations:** Cell & Molecular Pathology Laboratory, Department of Communication Sciences and Disorders, Northern Arizona University, Flagstaff, AZ 86011, USA; oneil.guthrie@nau.edu

**Keywords:** ear, hearing, sensorineural, genetic, treatment, gene editing, deafness, CRISPR-Cas9, congenital, hereditary

## Abstract

Gene therapy is a promising molecular approach for the management of familial hearing loss. This type of molecular therapy is the physical manifestation of genetic determinism—the notion that individual genes cause individual phenotypes. The current composition weaves through various branches of the biomedical sciences to uncover the molecular biologic premise for genetic determinism and the impetus behind gene therapy. Consequently, it is revealed that the underlying molecular biologic premise was scaffolded on successful observations from simple biologic assays that were devoid of the complexities of human disease biology. Furthermore, modern successful gene therapies are largely driven by commercial and academic incentives at the cost of scientific rigor. This poses several perverse challenges for patients, clinicians and the public at large. Issues concerning safety, efficacy, and ethics are far from resolved despite regulatory agency approvals, the media’s bias for gene therapy and the many lucrative investor positions. Lastly, the therapeutic claims regarding gene therapy are the most ambitious claims made within the hearing sciences. Therefore, scientists, clinicians, and patients must be equipped with the tools needed to appropriately consume and appraise such claims. These and other issues are also directly addressed, with the aim of providing a realistic sense of whether current human gene therapies are ready to be positioned within our routine clinical armamentarium against hearing loss.

## 1. Introduction

Hearing loss is the most common sensory deficit among humans and over 1.5 billion individuals currently have hearing loss which is expected to increase to one in four individuals by 2050 [1]. The global economic burden of hearing loss is estimated to be USD 1 trillion per year [1]. Remarkably, the world-wide disability-adjusted life-years for hearing loss exceeds 44 million years which supersedes that for other conditions that lead to morbidity and mortality, such as HIV/AIDS, falls, anxiety disorders, Alzheimer’s disease, self-harm, dietary iron deficiency, and interpersonal violence [2]. The clinical significance of hearing loss can be divided into five related domains [3]. (1) A social engagement domain that includes communicative disorders and differences along with academic achievement and social skills. (2) An environmental engagement domain that includes situational awareness, accidents, and injuries. (3) A physical activity domain that includes falls, frailty, and mobility. (4) A mental-status domain that includes neurologic, psychiatric, and psychologic sequalae. (5) An economic outcomes domain that includes the cost burdens associated with hearing healthcare, special education, and lost vocational productivity. Although hearing loss is highly heterogenous, the five domains of clinical significance can be applied to all types or classifications of hearing loss. Gene therapy has been positioned as a precision approach that could impact all five domains by directly addressing the underlying cause of hearing loss. For instance, if a given hearing loss is caused by a particular gene, then replacing the damaged gene with a new copy is believed to restore hearing function thus limiting the impact of the five clinically significant domains at both the individual/patient, and societal levels.

Familial (also known as hereditary) hearing loss (FHL) tends to occur among specific families and may manifest a range of audiologic characteristics (phenotypes) such as (but not limited to): severity (mild to profound), configuration (low, mid, high, etc.), type (conductive, sensorineural, mixed, etc.), laterality (unilateral and bilateral), symmetry (symmetric or asymmetric) and onset (congenital, prelingual, postlingual, etc.). Up to 91% of FHL exhibit an inheritance pattern where the hearing loss occurs within every generation or skip some generations [4]. FHL is particularly prevalent among individuals who exhibit congenital onset, bilateral loss, and a normal physical examination. This highlights the need for a thorough audiologic/otologic case history that includes the family history of hearing loss. FHL is a multivariate phenomenon, and environment is an important variable. Environment includes the sociotypes (bio-psycho-social factors) and ecotypes (niche environment) in which the family as a whole and/or sub-sets of the family are exposed. For instance, FHL can be as high as 72% in Middle Eastern families [4]. This highlights the need to interrogate case histories that includes family history of hearing loss against recent and past socio-environmental circumstances.

**Figure 1:**
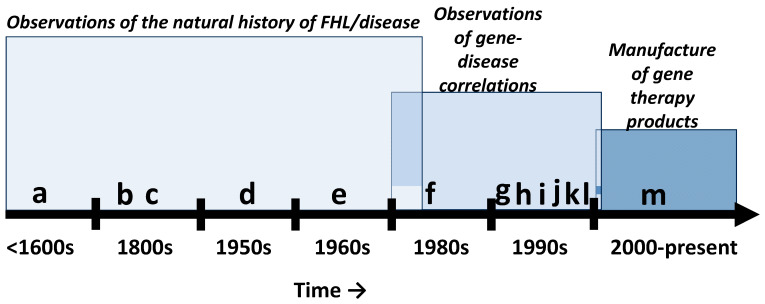
Familial hearing loss (FHL) milestones. (**a**) Observations of FHL in endogamous communities (e.g., Island of Martha’s Vineyard-GJB2/connexin 26). (**b**) Recognition of FHL risk factors such as consanguinity, familial diseases, environments, and behaviors. (**c**) Descriptions of various FHL patterns (e.g., those that could and could not be predicted by statistics) and associated syndromes (Usher, Branchio-oto-renal, Pendred, Treacher Collins, osteogenesis imperfecta tarda, etc.). (**d**) Correlation of audiometric characteristics with putative etiology (medical conditions, dominant vs. recessive FHL, etc.). (**e**) Further recognition of Mendelian and non-Mendelian patterns of FHL. (**f**) Characterization of DFNX2 (*POU3F4*, OMIM: 300039). (**g**) Characterization of DFNA1 (*DIAPH1*, OMIM: 602121). (**h**) Characterization of DFNB1 (*GJB2*, OMIM: 220290). (**i**) Characterization of pathogenic variants (e.g., within *GJB2*). (**j**) Mapping of syndromic genes (e.g., *USH2A*, OMIM: 608400). (**k**) In vitro studies on gene therapy (e.g., h*BDNF*, OMIM: 113505 delivered via herpes simplex virus to spiral ganglion explants). (**l**) In vivo studies on gene therapy (e.g., h*BDNF* delivered via herpes simplex virus to mice). (**m**) The introduction of commercial gene therapy products (e.g., AK-OTOF, AAV.103, OTOV-101).

Since the late 20th century, there has been a concerted effort to molecularize FHL (Figure 1) which has led to pseudo-synonyms such as monogenetic, polygenetic, genetic, Mendelian, etc. Targeted genome enrichment and massively parallel sequencing technology have evolved to become the most important tools used in comprehensive genetic assessments of FHL. Such technological achievements have demonstrated that human DNA sequence variations (changes in DNA) are correlated with FHL which has solidified a gene-centric view of the phenomena. For instance, sequence variants within the human *GJB2* (OMIM: 121011) gene is the most common correlate of FHL and nearly 7 of 10 (~70%) coding nucleotides within this gene will exhibit a variant (a change), while other deafness genes may exhibit 1 to 5 [5]. Therefore, it is believed that a powerful way of understanding FHL is to consider the underlying sequence variations in DNA (the gene) and DNA products such as RNA and protein (see Table 1). Furthermore, there is the belief that replacing the DNA (e.g., genes with sequence variations) will then restore hearing. Indeed, this was recently demonstrated in congenitally deaf children (age 1 to 8 years) who exhibited sequence variations such as c.2985C>A, p.C995* and c.5203C>T, p.R1735W within the *OTOF* (OMIM: 603681) gene [6,7]. Here, the children were administered a virus (vector) that contained the normal copy of the *OTOF* gene. This gene therapy strategy is called replacement because it was assumed that the children’s sequence variations lead to loss-of-function (LOF) for the gene products (RNA and protein); therefore the normal *OTOF* gene was expected to replace the lost function.

Scientists, audiologists, otologists, and otolaryngologists are principal members of the clinical team involved in the management of FHL and must have a working understanding of the nomenclature used to describe human sequence variations (Table 1) and have access to relevant supporting resources (Table 2). Today close to 1 million sequence variants have been detected within genes that correlate with hearing loss and over 8000 of these variants are in silico predicted to be clinically relevant [5]. Therefore, gene therapy has emerged as a sophisticated solution and a direct strategy for replacing genes with LOF sequence variations. Interestingly, LOF variants have been implicated in almost all audiologic/otologic sequalae (even routine acquired forms of hearing loss) and gene therapy is considered a universal treatment approach. The two discussions below provide a modern, balanced, and realistic appraisal of gene therapy. First, an historical context is provided which considers the different phases of scientific developments that ultimately led to gene therapy. Second, researchers, clinicians, patients, and other stakeholders are introduced to practical ways of thinking about and evaluating gene therapy claims.

## 2. Historical Context

The modern practice of gene therapy is based on two fundamental assumptions. First, genes cause diseases. This one-cause one-outcome (binary) way of thinking evolved from an ancient paradigm that originated within the chemical sciences. Second, the manipulation of genes will cure diseases. This binary way of thinking reduces human disease complexity to one magic bullet per disease and originated from biological demonstrations on viruses, fungi, bacteria, and cell lines. Here, primordial biologic assays revealed that phenotypes (biologic features) were transferred from one generation to the next and this inheritance was associated with genes. Therefore, correlations and associations between genes and phenotypes were generalized to mean cause (genotype) and effect (phenotype). Such gene-centric (genes determine outcomes) way of thinking paved the way for the current clinical practice of using genes to treat diseases. The discussion below provides an historical account of the two fundamental assumptions that underly modern gene therapy.

**Figure 2:**
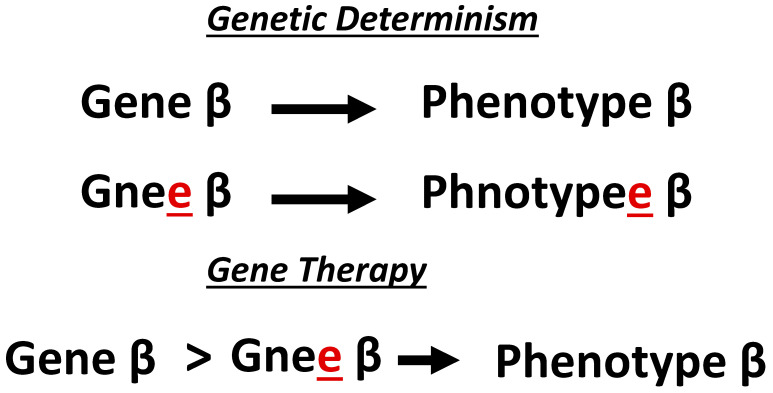
Gene therapy is the practical manifestation of genetic determinism. Genetic determinism is paradigmatic of the dualism paradigm, where a single gene (e.g., gene β) is believed to cause a specific phenotype (e.g., phenotype β) and if there is a sequence variant in the gene (e.g., gnee β) then the phenotype (e.g., phnotypee β) will be affected. Therefore, gene therapy seeks to obtain the correct phenotype by overcoming the sequence variant.

Dualism is a superannuated paradigm that views the natural and supernatural world as binary. Concepts such as genotype–phenotype, nature–nurture, ying–yang, mind–body, good–evil are all dualistic in nature. Prior to the 17th century, three classes of compounds were known: vegetables, animals, and minerals [9]. In the 1700s, chemists determined that vegetables and animals were composed of carbon and hydrogen, but some nitrogen and phosphorus [9]. This supported a dualistic worldview of organic (living things like vegetables and animals) vs. inorganic (nonliving items like minerals) laws of nature. However, in the 1800s chemical experiments revealed that organic substances (e.g., urea) could be derived from inorganic substances (e.g., ammonium cyanate). Furthermore, substances (e.g., acetic acid) could be synthesized from several elements. These and other observations convinced some chemists to partially abandon a dualistic nature of the world. Biologists ultimately coopted dualism as the prevailing paradigm of the life sciences. One biological formalization of dualism is a sub-paradigm called, genetic determinism, the notion that a single gene determines (causes) a single phenotype (Figure 2). For instance, the belief that genes cause human diseases (genotype–phenotype) is representative of a genetic deterministic way of thinking.

Today, genetic determinism can be observed in all the major branches of the biomedical sciences. Indeed, the concept of genetic determinism is the prevailing influence on current therapeutic strategies. For instance, across the biomedical sciences and regardless of the molecular pathology, clinicians and scientist predominantly pursue gene replacement as the dominant gene therapy strategy [10]. In gene replacement, it is assumed that a variant in a gene caused a particular human disease (the notion of genetic determinism). This is the reason why current molecular diagnoses deploy high-throughput technologies that are focused on sequencing whole-genomes or targeted motifs within the genome so that gene variants can be detected. Furthermore, replacing the gene harboring the variant with a normal (non-variant) copy is believed to restore normal protein function to cure diseases (yet again, the notion of genetic determinism). Gene therapy is the practical manifestation of genetic determinism. In gene therapy, a vector such as a virus is used to deliver a therapeutic gene to treat a specific disease. The history of gene therapy can be divided into three phases that are not necessarily linear in time: basic science, pre-commercialization, and commercialization. The discussion below will weave through different branches of the life sciences to reveal how we ended up with the genetic deterministic practice (genes cause diseases and gene manipulations are needed to fix diseases) of gene therapy.

### 2.1. Basic Science Phase

Inherited Traits: For thousands of years, farmers and others have demonstrated that selective breeding within species (sheep, humans, plants, etc.) produced desired and undesired traits. Such traits were not random and could be inherited from either parental lineage. The patterns of trait inheritance were sometimes predictable by statistical computations (e.g., polydactyly) and some patterns followed what we now refer to as autosomal dominant, autosomal recessive, sex-linked, and mitochondrial. Inherited traits were known to be associated with parental germline as well as other factors, exposures, and behaviors. In farming, it was believed that blood was the biologic mechanism of trait inheritance, but the farmers also knew that environment was an important variable. Others believed that small particles (in plants and germ cells) were derived from various parts of the adult body and these particles could then reproduce the whole body of an embryo. In the 1800s, plant biologists adopted what was already known by farmers (and others) to construct what is now called Mendelian genetics. Additionally, cell biologists posit that nuclear chromosomes contained the small particles responsible for hereditary traits. Also in the 1800s, biochemists were characterizing the nucleus within cells, chromosomes within the nucleus, nucleic acids (DNA and RNA) within chromosomes and the five nucleotide bases within nucleic acids, namely guanine (G), cytosine (C), adenine (A), thymine (T), and uracil (U). Although the locus and constituents of DNA were defined in the 1800s, its function was still debatable until the discovery of the transforming principle.

Transforming Principle: The transforming principle is the process of changing an organism’s phenotype with foreign DNA either via transfection (non-virus mediated) or transduction (virus mediated). It was important for modern gene therapy for two reasons. First, it suggested that DNA is associated with biologic traits which was then generalized to mean that DNA caused biologic traits. Second, it suggested that viral and non-viral vectors could be packaged with therapeutic DNA (called transgene) and subsequently delivered to a host.

Microbiologists revealed that DNA was the substance of trait inheritance by demonstrating transfection [11]. In these demonstrations, non-virulent bacteria were transformed into virulent bacteria. For instance, the filtered intracellular contents of the virulent S- (smooth form of) type II-pneumococcal bacteria was superfused to growing culture of non-virulent R- (rough form of) type I-pneumococcal bacterial and the result was a transformation to virulence [12]. Such demonstrations suggested that an organic transforming substance caused the transformation. Although the underlying substance was unknown at the time but speculated to be proteins, the transforming substance could be alcohol precipitated (today it is routine practice to alcohol pellet DNA out of solution). This precipitating substance was later identified as DNA [13]. Additionally, from the 1930s to 1940s, biochemists confirmed that DNA was localized within chromosomes, and DNA X-ray diffraction patterns were resolved.

Microbiologists also outlined the processes of conjugation and transduction [14]. Conjugation is the observation that certain bacteria transfer DNA by mating. Transduction is the observation that bacteriophage (virus that infect bacteria) can transmit DNA between two bacteria. For example, a fine glass filter was used to separate drug-resistant Salmonella from wild-type Salmonella yet the two inherited each other’s traits because bacteriophage was serving as vector by transporting DNA between them. This observation provided a rationale for why bacteria of different strains can develop antibiotic resistance. But more importantly, bacteriophage proteins were not transferred between the bacterial strains, only DNA. This led to the notion that viruses could serve as vectors to deliver DNA [15]. Furthermore, microbiologists revealed that inheritance could occur not only from DNA to RNA but also from RNA to DNA [16]. For instance, the Rous Sarcoma RNA virus (RSv) which harbored a variant could transfer the variant to chicken cells (RNA→DNA) via chromosomal insertion, then the chicken cells could transfer the variant to the next generation of RSv (DNA→RNA). Also in the 1950s, biochemists again deployed X-ray diffraction to reveal crystalline (A-form) and paracrystalline (B-form) DNA structures [17] and molecular biologists uncovered the triplet code [18]. This is the three base code system (called codons) within DNA that is used to make the twenty amino acids that constitute proteins. Taken together, basic science research on viruses and bacteria revealed the function and structure of DNA which ultimately allowed for the development of genetic engineering tools.

Genetic Engineering: Genetic engineering refers to gene modifications. Exposure of the ascomycete fungi (neurospora) to X-rays produced gene modifications that supported a one-gene one-enzyme concept, where modifications to specific genes correlated with specific phenotypes associated with pyridoxine (vitamin B_6_) synthesis [19]. Restriction and ligation enzymes were also characterized and revealed to be gene modification tools [20,21]. A restriction enzyme is a special protein that cleaves DNA sequences at precise locations while a ligation enzyme is a protein that joins (ligates) DNA sequences. Therefore, a restriction enzyme can be used to remove a gene from a specific sequence of DNA (called restriction digest) then a ligation enzyme (called a ligase) can be used to join a new gene to the same surrounding sequence of DNA. A sequence of DNA that has been manipulated in these and other ways is called recombinant DNA (rDNA). Restriction and ligation enzymes are the foundations of gene manipulation technologies, and they are used to construct recombinant viral vectors, which are used in gene therapy today. For instance, natural pathologic genes within a sequence of viral DNA are replaced with a therapeutic transgene (new gene). Additionally, cell biologists demonstrated that sequence variants within the D98S human cell line could be rescued via transfer of DNA from cell lines without the variants and the transferred DNA could be inherited by daughter cells [22]. Lastly, it was observed that viral DNA integrates into host cell genomes, which is the basis of insertional mutagenesis [23]. Insertional mutagenesis (a major toxic limitation of viral vectors today) is the incorporation of foreign materials, such as vectors, into the host (e.g., patient) genome (both nuclear and/or mitochondria) which then interferes with the normal functions of the host genomes.

The early DNA research efforts described above are directly connected to current gene therapy applications. For instance, they helped to solidify a gene-centric view of biology, where observed traits are explicitly directed by DNA and when these traits become abnormal then DNA manipulation could solve the problem. These early DNA research efforts were highly successful at demonstrating the biological importance of DNA because they were conducted on simple biological systems (viruses, bacteria, cell lines, etc.) that were devoid of the complexities associated with human disease biology. Nonetheless, research on inherited traits, the transforming principle and genetic engineering, ultimately ushered in the pre-commercialization phase of gene therapy.

### 2.2. Pre-Commercialization Phase

Gene tracking: DNA marking techniques were developed to track the movement of cells [24]. Here, cells were extracted from an animal then transduced with a virus carrying a gene-of-interest then the cells were re-introduced to the animal. This specific process is called ex vivo (outside the body) gene therapy and it was eventually used on humans. For instance, a recombinant retroviral vector carrying a marker gene coding for resistance to neomycin was used to transduce human tumor-infiltrating lymphocytes (TIL) that contributed to metastatic melanoma [25]. The TILs were extracted from the patients, cultured, and then transduced with the neomycin resistance marker gene. The re-introduction of transduced TILL to the patients allowed for tracking the survival and biodistribution of the cells during immunotherapy for metastatic melanoma. Such gene tracking efforts were successful because genes are indeed one variable within a multivariable process that produces proteins within cells. The success of gene tracking, first in animals and then in human patients paved the way for early human gene therapy.

Setbacks: Setbacks to early gene therapy trials ranged from poor scientific rationale and procedural over-sight to more serious outcomes such as insertional mutagenesis, severe immune responses, and malignancies that ultimately led to deaths. Two girls with urea cycle disorder (genetic arginase deficiency) were infected with wild-type Shope papilloma virus [26]. It was hypothesized that the viral genome naturally codes for arginase (the enzyme that facilitates the final step in the urea cycle). However, the therapeutic approach produced little improvement in arginase levels among the patients. Through sequencing the Shope papilloma viral genome, we now know that the wild-type virus does not naturally contain the arginase gene. A multi-site clinical trial was conducted to correct hemoglobulin gene variants in extracted bone marrow stem cells from patients with β-thalassemia. However, the trial was discontinued because it failed to secure full IRB approval [27]. This has now influenced current clinical practice by ensuring that all gene therapy trials receive coordinated institutional approval and oversight, even for multinational trials. However, the β-thalassemia trial and other previous trials failed to ensure that all participants in gene therapy trials are properly consented regarding life threatening risk-factors from the therapy (this continues to be the case even for gene therapy trials performed today). For example, an 18 year old male (Jesse Gelsinger) who suffered with a rare metabolic disease died in a University of Pennsylvania clinical trial, where he received an adenoviral vector (Ad5) carrying a normal copy of the ornithine transcarbamylase gene [28]. His death was attributed to gene therapy-induced cytokine storm (severe immune reactions) resulting in multi-organ failure and death within four days of the therapy. Furthermore, up to 25% of patients within a European clinical trial developed (within 2.5 to 5 years post-therapy) T-cell leukemia as a result of transgene activation of oncogenes (genes involved in the cell cycle that fuel cancer) and one patient ultimately died [29]. Even the most definitive examples of gene therapy cures were plagued with subsequent development of cancer [30,31]. Therefore, a *conditio sine qua non* for any gene therapy (even inner ear) is long-term (in years) monitoring for subsequent adverse events.

Several important lessons were learned from the setbacks but those associated with immune responses and oncogenesis deserve additional emphasis. The human immune system has evolved to react to foreign invaders such as viruses and other non-viral particles; therefore the use of a viral gene therapy must be expected to elicit an immune response. Current viral gene therapy trials typically conduct crude (e.g., blood and CSF) measures of immune responses which result in misleading conclusions regarding the therapy’s potential to induce an immune response [32]. We now know that gene therapies may illicit tissue specific immune responses that evade current measures of immunomonitoring. For instance, it is widely believed that the mammalian inner ear is immunoprivileged due to the blood–labyrinth barrier; however, this is incorrect and gene therapy will illicit both innate and adaptive immune responses in the inner ear [33]. This is particularly important because the immune response may induce localized (within target and non-target tissues) cytotoxicity. Virtually all components of modern gene therapy (capsid, viral DNA, etc.) can elicit an immune response. Even the presence of the therapeutic transgene within a given tissue can illicit an immune response. This means that the efficacy of the therapeutic transgene can be quenched by host-specific immune responses and current clinical trials must verify long-term (in years) expression of the target gene within the target tissue for each patient. This is particularly relevant because immunomodulatory drugs (corticosteroids, imlifidase, proteasome inhibitors, calcineurin inhibitors, mycophenolate, rapamycin, rituximab, etc.) will never reliably protect patients and such drugs add to the problem by introducing their own toxicities, side-effects, interactions, and confounds. These were lessons learned the hard way in the hemophilia field, the neuromuscular disease field, and several other biomedical fields.

With regard to oncogenesis, consideration must be given to the fact that gene therapy will naturally produce artificial expression patterns of the therapeutic transgene (this will occur even with tissue specific promoters and enhancers). Such artificial expression will affect multiple cell-signaling cascades that may drive pathologic outcomes such as oncogenesis [34]. Indeed, gene therapy-induced oncogenesis occurs years after administration of the therapy. Therefore, modern gene therapy clinical trials cannot claim to be safe in the absence of years of safety monitoring following therapy administration.

Successes: There were several examples of successful outcomes from gene therapy. For instance, the case of adenosine deaminase deficiency-severe combined immunodeficiency where two children were treated with a recombinant retroviral vector to deliver a normal copy of the adenosine deaminase (*ADA*) gene into their T-cells [35]. The treatment lasted for two years and resulted in the normalization of T-cells, humoral immune system, and other cellular responses. Furthermore, the retroviral vector and *ADA* (OMIM: 608958) gene expression persisted within the T-cells. However, this study was confounded by the fact that one patient was also receiving enzyme replacement therapy with polyethylene glycol adenine deaminase [36]. Success in gene therapy trials depends on how one critically assesses the nature of the circumstances leading to success. For instance, almost all successful gene therapy trials (both in the past and today) deploy study designs that specifically bias the outcomes in favor of the therapy and thus lacks scientific rigor [37,38]. Nonetheless, the success of the pre-commercialization phase directly paved the way for the commercialization phase of gene therapy.

### 2.3. Commercialization Phase

Accessibility and market potential: Research and the development of gene therapies are typically initiated in academia (independent scientific labs) and then progress to pharmaceutical companies following the establishment of commercial agreements. Such academic/commercial partnerships generally create a conflict of incentives where academicians are incentivized to pursue tenure, promotion, publications, and grant funding while the commercial partners are incentivized to pursue profits over scientific rigor. Such divergence in incentives help to establish disparities in patient accessibility to gene therapies and the commercial potential of the therapies. Indeed, the scientific literature has delineated at least seven hurdles that perpetuate disparities between patient accessibility of therapies and market potential [37,38].

(1) Affordability encompasses the excessively high price for individual gene therapies and the limits of insurance coverage. Additionally, long-term medical costs of the morbidities that develop over years after gene therapy is known to be a highly significant contributor to affordability.
(2) Assessment-of-value encompasses the perceived value for individual patients and their families, as well as perceived value within individual countries, cultures, and healthcare systems/agencies.
(3) Development-of-therapy relates to the fact that all manufactured gene therapy products yield some degree of uncertainty in manufacturing precision/chemistry. This includes patients treated with vectors that mistakenly omit the therapeutic transgene and vectors with incorrect, misaligned, or mutated transgenes and/or vector DNA sequences. The penultimate consequence of these unintended manufacturing uncertainties is a high-level failure rate (in terms of efficacy and safety) in clinical trials which again limits patient accessibility.
(4) Ethical/social factors encompass negative religious, cultural, political, and socioeconomic beliefs and misinformation against gene therapy. Furthermore, disparities in costs and standards-of-care within and between countries creates heterogenous barriers for different populations and perpetuate notions of “*treatment tourists*”.
(5) Evidence generation relates to the fact that most gene therapy clinical trials do not meet established scientific standards for credible evidence which then limits patients’ access to therapies. For instance, clinical trials and the data produced from the trials need to exhibit specific characteristics to be considered credible, such as (but not limited to) randomization of patients/independent sampling; inclusion of a control arm/group; double/triple-blinding; placebo; statistical power; constrained multiplicity and parametric data homoscedasticity and Gaussian distribution. Interestingly, gene therapy researchers and clinicians typically justify the absence of the above standards with two colloquial excuses. One is that “*the field is too new*”; however, human gene therapy can be traced back to the Shope papilloma virus in 1973 (>5 decades old). The second is “*gene therapy research is just too difficult*”. Yet across the biomedical sciences, difficulty has never been an appropriate excuse for sloppy research. The consequences of lowering scientific standards for gene therapy research is exemplified by many perverse and dire examples in the literature. In the X-linked myotubular myopathy field, lowered standards resulted in a death rate that increased to 30% with the more recent death of another patient (NCT03199469). In the Duchenne muscular dystrophy (DMD) field, cardiovascular field, and several other fields, years of studies with lowered scientific standards perpetuated the notion that gene therapy can cure DMD, cardiovascular diseases and other genetic diseases [39,40]. However, when consortiums of institutions and researchers finally conducted scientifically rigorous studies, it was revealed that the gene therapies were never cures. This ultimately led to decades of wasted resources, time, unnecessary toxic exposures, and untimely deaths for study subjects.
(6) Operational implementation encompasses barriers endemic to both patients and healthcare providers. For instance, patients need to have access to a hospital equipped with specialized pre- and post-therapy administration protocols, methods, facilities, and personnel. This limits access for many patients and burdens others who would have to travel great distances. Healthcare providers need to be formally trained to provide the specialized patientcare needed before and after therapy administration. Such formal training includes specialized safety and rescue procedures that are bespoke to the acute and chronic toxic outcomes for each gene therapy product as well as training on managing unintended deaths and life-threatening adverse events from the therapy. Furthermore, the high administrative burdens (added medical record keeping, proper patient consent, health insurance forms, etc.) further compound the situation, which all serve to limit the number of providers willing to serve patients.
(7) Regulatory hurdles encompass the philosophies, practices, and procedures of regulatory bodies such as the US Food and Drug Administration (FDA), European Medicine Agency (EMA), Health Canada, Russian Ministry of Health, and the State Food and Drug Administration of China (SFDA). Here, a major barrier to patient access is the disparities between regulatory bodies. For instance, gene therapies approved by the EMA may not receive approval from the FDA and others. Additionally, approval of a given therapy does not mean widespread use or acceptance by patients and healthcare providers. Furthermore, the level of safety and quality control is different among the regulatory bodies, and some allow for parallel (less regulated) pathways to achieve therapy approval. Even more frustrating is the fact that disparities exist within regulatory bodies, for example, member states within the European Union. Interestingly, regulatory bodies within individual countries can be segregated by whether they prioritize clinical benefit from gene therapy, costs associated with gene therapy, or both.

Non-auditory products: Many companies have developed gene therapy products (Gendicine™, Oncorine™, Cerepro^®^, etc.) for a large variety of medical conditions (Leber congenital amaurosis, β-thalassemia, SCID-X1, etc.). The majority of such therapies are used to treat cancer followed by genetic disorders and then infection [41]. Hematological cancer accounts for almost half of the gene therapy products that target cancer while metabolic disease accounts for most genetic disorders that are targeted. For infectious diseases, the majority of products target HIV. Most products use viral vectors, such as lentivirus, adenovirus (Ad), adeno-associated virus (AAV), and retrovirus. Lentivirus and retrovirus are used for ex vivo gene therapy while Ad and AAV are used for in vivo (inside the body) gene therapy. Ultimately, hundreds of gene therapy products have now been approved (FDA, EMA, SFDA, etc.) and hundreds more are currently waiting for approval. Both existing and pending products are indicated for an impressive variety of medical conditions, and it would be fair to conclude that *we currently reside in the era of gene therapy*.

Auditory products: Given that the hearing loss market is valued at USD 9 billion and hearing loss affects nearly 20% of the global population, it was only a matter of time before companies entered the auditory gene therapy space (Figure 1). Among the most interesting hearing loss products was CGF166 (NCT02132130) by Novartis Pharmaceuticals. CGF166 was composed of a recombinant Ad5 vector containing the therapeutic *ATOH1* (OMIM: 601461) gene. The *ATOH1* gene is the most well studied across the hearing sciences and hearing researchers considered this gene to be, “*both necessary and sufficient*” to regenerate cochlear hair cells [42]. Indeed, *ATOH1* was supposed to save us from all forms of hearing/auditory conditions and promised to revolutionize stem cell therapy and hair cell regeneration (akin to a god gene). Although the gene was highly successful in pre-clinical (animal models) studies, it ultimately failed in human trials and some patients even exhibited worst hearing thresholds due to the gene therapy. There were many reasons for this failure, and they range from the underlying molecular and cellular biology to the systemic biases imbued by the researchers in this field [8]. However, it is worth noting that the researchers consistently reduced the complexity of intricate biologic phenomena (hearing, hearing loss, and restoration of hearing) to a single gene (quintessential examples of genetic determinism/dualism). Consider that the patterning, morphology, and physiology of mammalian cochlear hair cells are dependent on multiplicative and multifunctional signaling cascades that are in term choreographed by several types of auditory and non-auditory cells, tissues, organs, systems, and environments [8]. Therefore, it was unrealistic to expect that the *ATOH1* gene alone could orchestrate the necessary biologic constraints within the context of the interaction between the disease-state and the unique physiology of each patient within the clinical trial. Indeed, the gene-centric purview that perpetuated the *ATOH1* gene movement was made possible by willfully ignoring 160 years of systems biology in general (circa 1865-*Introduction a L’étude de la Médecine Expérimentale*. Flammarion, Paris) and 64 years of inner ear systems physiology in particular (circa 1961-*Neue Aspekte zur Biologie und Pathologie des Innenohres*, Arch. Ohren-, Nasen- und Kehlkopfheilk. 178, 1–104).

The current vogue is the *OTOF* gene and there appears to be several commercial products designed to treat defects in this gene. The FDA has even granted Rare Pediatric Disease Designation to at least one product (Sensorion, OTOF-GT). However, the current movement behind the *OTOF* gene as a cure for human hearing loss is poised to eventually yield the exact fate as that of the *ATOH1* gene movement for the exact reasons discussed above (also see discussion below). Pre-clinical research on *ATOH1* and *OTOF* are always conducted on animal models rather than model animals therefore the research findings will never reliably translate to clinical forms of hearing loss [8]. Additionally, viral tropism (the ability to infect) in one animal model due to molecular permutations of transmembrane receptors, unique circulation of diffusible signaling molecules, and their interactions will never consistently translate to other strains of the same species or between individual human patients. These and other basic preclinical variables are often overlooked which culminate in high failure rates in clinical trials as exemplified in the recent failure to regenerate human hair cells with a gamma-secretase inhibitor [43]. Nevertheless, commercial interest in gene therapy for hearing loss has intensified in the last 20 years, with an ever-expanding number of new products. Most of these products use viral vectors, in particular the AAV to target FHL.

The use of AAV is facile given the multitude of delivery routes for administering the vector into inner ear fluids. These delivery routes include (but not limited to), transtympanic, round window membrane, stapedotomy, cochleostomy, and canalostomy. The most popular appears to be through the round window membrane. Here, the therapy is injected through the round window and into cochlear fluids while a fenestration is made at the stapes footplate to facilitate perfusion of the therapy throughout the inner ear [6]. It is important to recognize the symbiotic relationship between the inner ear and the rest of the body. Indeed, viruses such as AAV can easily escape the cochlea to infect the brain [44]. Furthermore, after injection into inner ear fluids, AAV may rapidly spread to blood which causes an increase in neutralizing antibodies against AAV and/or the therapeutic transgene [45]. This may result in two important consequences. First, the neutralizing antibodies may seek out and destroy the gene therapy which means that gene therapy trials must assess both short and long-term efficacy. Second, the risk of cytokine storms and systemic organ toxicity is a real possibility even after inner ear fluid injection.

Current and future gene therapy products for hearing loss are poised to deploy the AAV vector and the genetic engineering of these vectors can only be described as remarkable. For instance, the AAV vector has a limited capacity to carry therapeutic transgenes (packaging capacity of only 4.7 kb) because inner ear therapeutic transgenes are often too large (6 to 10.6 kb). To rectify this limitation, the therapeutic transgene is spliced into two parts (5′ and 3′ coding sequences), where one part is carried by one AAV vector while the other part is carried by another AAV vector. This dual-AAV approach is expected to reconstitute the full-length therapeutic transgene at the DNA, pre-mRNA, or protein levels after the vectors have entered cochlear cells, such as hair cells. Indeed, this duel-AAV approach is now the standard in the industry and represents a triumph for inner ear gene therapy. However, there are serious concerns with this approach.

**Figure 3:**
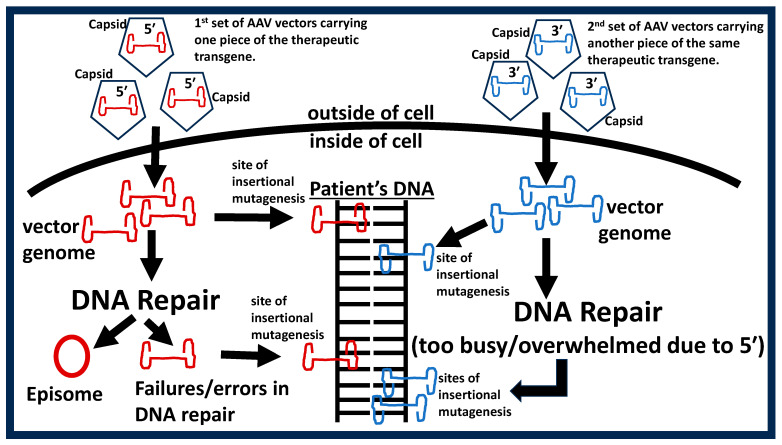
Dual-AAV vectors increase the risk of insertional mutagenesis. The therapeutic transgene is spliced into two pieces (5′ and 3′) and each piece is carried by separate AAV vectors (see red vs. blue in figure). After the AAV vectors enters a given cell, the capsid (protein coat then encircles the vector genome) is removed, and the single stranded vector genome is revealed. Some of these single stranded vector genomes will insert themselves in the host (e.g., patient’s) DNA (both mitochondria and nuclear DNA) until DNA repair factors are available to transform the single-stranded vector genomes into circular double-stranded particles called episomes (episomes are considered safe but this is not entirely true). However, due to the error-prone nature of DNA repair, and since DNA repair efficiency is different between cells (even cells of the same type), there will be a fraction of the single-stranded DNA that will eventually insert themselves within the host DNA. These various opportunities for insertional mutagenesis are at least doubled with the use of dual-AAV because the second family of vectors (blue in figure) will also induce insertional mutagenesis in a similar manner. Furthermore, the second family of vectors will likely not have the benefit of DNA repair factors since the first family of vectors (red in the figure) have coopted all or most of the available DNA repair factors (a process called molecular hijacking) which leads to even more insertional mutagenesis.

When AAV enters a cell, it releases its single-stranded genome which is converted to circular double-stranded genome by the host-cell’s (e.g., hair cell) internal DNA repair pathways. Here, the function of the host-cell’s DNA repair pathway is essential because failure of this pathway would result in a failure in producing double-stranded vector genomes which would then result in insertional mutagenesis. A long history of research has characterized the DNA repair pathways within the inner ear and such research has shown that different cells exhibit varying capacity for DNA repair [46,47,48,49,50]. For instance, hair cells and spiral ganglion neurons at the base of the cochlea exhibit delayed, slowed, and sometimes inefficient DNA repair responses [51,52,53]. This means that the basal cochlear coil could be prone to tumors due to single-AAV gene therapy, and this would be exacerbated with dual-AAV gene therapy. Indeed, it is known that AAV vectors can insert themselves within normal genes, proto-oncogenes, tumor suppressors, ribosomal DNA sequences, CpG islands, palindromic sequences, transcription initiation sites, etc. [54]. Any one of these could contribute to cancer development, which is a critical but often overlooked consideration (see Figure 3). Therefore, AAV inner ear gene therapy may need to be complemented with molecular therapies that have been shown to increase DNA repair capacity within the inner ear [55,56,57,58,59,60].

The risks of insertional mutagenesis, oncogenesis, deaths, and localized and systemic toxicities are major limitations to the universal acceptance of gene therapies. Therefore, it is reasonable to consider strategies to mitigate these risks. Unfortunately, no satisfactory strategies have emerged. This is based, in part, on the fact that morbidities and mortalities associated with gene therapies typically receive little to no attention compared to findings that demonstrate a positive effect (systemic publication bias). Furthermore, gene therapy articles written by gene-centric researchers take great care to espouse the notion that gene therapies are both safe and efficacious. Hidden from the reader are the many caveats disclosed in the current composition (see discussions above and below). Rather than ignoring these caveats, a more fruitful approach would be to position them at center stage to leverage the collective expertise of the biomedical enterprise in resolving each caveat. Only then can reasonable strategies emerge to mitigate the potentially life-threatening risk factors and only then can true innervations be realized.

Hearing healthcare clinicians face several ethical dilemmas that include but are not limited to the following.

1. Are the risks of morbidity and mortality associated with gene therapy reasonable in the context of deafness/hearing loss?
2. Can clinicians really claim that the therapy is safe and effective, given the current publication bias and poor-quality clinical trials (see discussion below)?
3. Given current hyperbolized claims regarding gene therapy (see discussion below), are clinicians adequately consenting their patients before gene therapy trials?
4. Given the well-known efficacy of hearing aids and cochlear implants, is it reasonable for clinicians to bias counseling towards gene therapy or position gene therapy as having similar levels of efficacy as hearing aids and cochlear implants?
5. Should clinicians support parents who would rather wait for gene therapy to become available rather than manage the hearing loss with cochlear implants?

Unfortunately, these and other ethical dilemmas have not been explicitly addressed in the hearing healthcare community and evidence-based answers to the above dilemmas are limited. What is needed are community discussions that yield *Marquess of Queensberry like-rules* to govern clinical perspectives and applications of gene therapy.

In summary, dualism is the overarching paradigm that led to genetic determinism (gene to phenotype way of thinking) and ultimately the practice of using genes to affect phenotypes or what we now refer to as gene therapy. The road to gene therapy can be divided into three phases. The basic science phase was replete with research demonstrating that DNA was inherited from generation to generation and codes within the DNA (called genes) carried instructions for making proteins (gene products) that ultimately contribute to phenotypes. Indeed, alterations to the DNA code (genetic variants) were correlated with inherited traits (good, bad, or indifferent). Furthermore, it was demonstrated that viruses could be used as DNA vectors. Therefore, the Basic Science Phase provided the scaffold needed for the Pre-commercialization Phase of gene therapy. Here, individual investigators identified a problem (e.g., a disease-state) and then pursued gene therapy as a possible solution. The success from this phase heralded the Commercialization Phase of gene therapy. Given the potential to garner up to USD 4.25 million per dose (the world’s most expensive drugs), the Commercialization Phase is vitiated with an impressive variety of gene therapy products earmarked for almost every disease-state within the biomedical sciences. FHL is simply one of the disease-states that have attracted the attention of intrepid investors.

## 3. Scientist, Clinician and Patient Appraisal of Gene Therapy Claims

Over 2000 years ago, the popular hearing loss therapies included (but not limited to) goat urine, ant eggs, and red lead [8]. Given what we have learned over the past 2000 years, we are in a privileged position to properly appraise such therapies. Indeed, it seems silly that people would use such therapies because it is now obvious that they could not work, and some may even be detrimental. However, we should keep in mind that such ancient therapies were popular because there were many individuals who showed some level of hearing recovery (even total recovery) after a given therapy [61,62]. This is possible because of confounding variables that include (but not limited to) placebo effects, patient selection bias, and temporary or fluctuating hearing loss. Instead of urine, eggs, and lead, today we have gene therapy, stem cells, and hair-cell regeneration. There is no doubt that there will be many individuals who will also show some level of hearing recovery (even total recovery) with these modern-day therapies. Therefore, it is enticing to wonder how silly our modern-day therapies will seem to future clinicians and patients 2000 years from today.

Arguably, one of the most exciting developments in the hearing and hearing loss fields was the recent demonstrations that *OTOF* gene replacement therapy could recover FHL. These demonstrations were particularly important because no prior research had shown success in humans. This important discovery was followed by an impressive amount of media attention (*The New York Times*, *abcNews*, *NPR*, etc.) and became the topic of conversations at almost every professional conference (AAS, ARO, etc.). The stock of the parent company (Eli Lilly- NYSE: LLY) behind one of the therapies finally rose to a buy zone (zone in which investors can buy into or add to positions in stocks). The marketing success of *OTOF* gene therapy has been challenging for some clinicians who have parent-clients who are questioning whether they made a mistake in choosing cochlear implants for their children, since they could have simply waited for the gene therapy. Other parents want to forgo cochlear implants for their babies in hopes of pursuing gene therapy in the future. Given that a delay in auditory habilitation would have significant consequences for speech and language development, *how should professionals manage these and other concerns around gene therapy*? At the center of this issue is the fact that scientists, clinicians, and patients are consumers of the many research articles and media reports that proclaim that gene therapy recovered hearing. Therefore, scientists, clinicians, and patients will need to have tools for properly appraising research articles and media reports.

**Figure 4:**
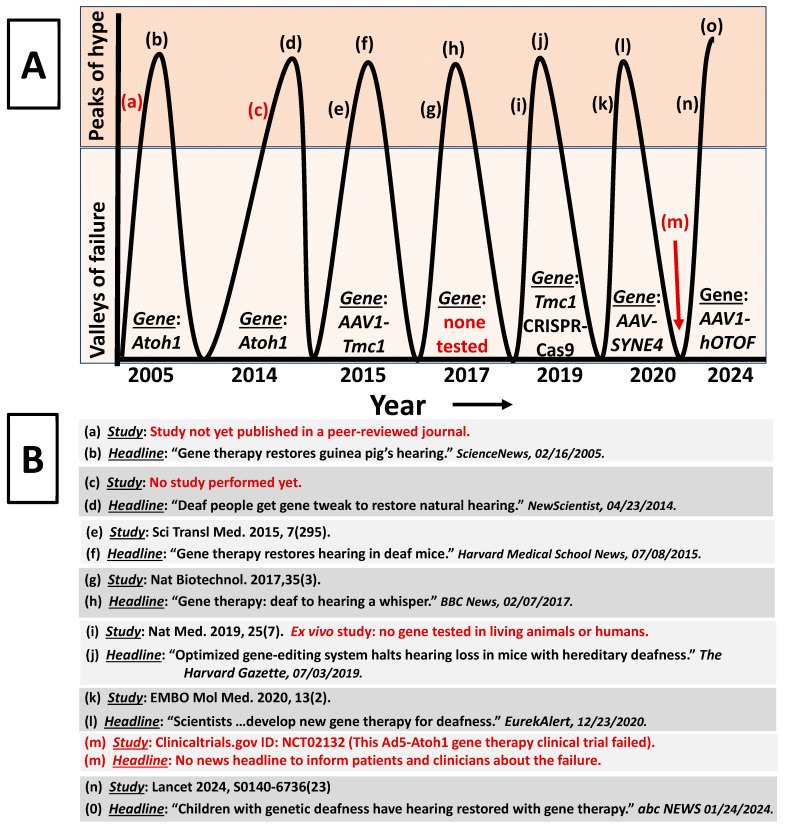
Periodicity of gene therapy claims. Panel (**A**) reveals that the increase in excitement (hype) over gene therapy is often fueled by a particular study followed by news headlines. However, there are instances where no study was published in a peer-reviewed journal, yet the headlines presuppose a successful future outcome. Additionally, there is an instance where no gene was studied, yet the news headline about the study proclaimed gene therapy success. Further, there is a study that did not even use a living animal or human, yet the news headline about the study claimed successful treatment of hearing loss. Interestingly, when a gene therapy fails, there appears to be little or no news headline about the failure (no hype). Panel (**B**) reveals the studies and news headlines from Panel (**A**). Red text in each panel indicates concerning scenarios. Reference cited [6,65,66,67,68,69,70,71,72].

As a first approximation, it is important to realize that claims about hearing recovery due to gene therapy are not new and such claims have spanned two decades now (Figure 4). Based on research articles and news reports, the gene therapy cure for hearing loss has been discovered several times in the past. In Figure 4A, we observe that there is a prominent trend of peaks and valleys, where the peaks are the periods when gene therapy researchers and writers of news reports tend to hyperbolize the results of a given research. This hyperbolism then captures the attention of clinicians and their patients and ultimately perpetuate more investments from investors and more grant funding for researchers. Interestingly, in the valleys within Figure 4A are the periods when gene therapy researchers and writers of news reports tended to be more constrained in their writings. These valleys are catacombs where gene therapy failures, bankruptcy, and company devaluations are buried from the public. Therefore, both clinicians and patients are unaware of the true successes and failures of gene therapy (also true for stem cell and hair cell regeneration therapies). FDA approval is frequently referenced as an elite standard for safety and efficacy for therapies. However, the *dirty little secret* is that the FDA has a track record of approving unsafe and inefficacious therapies [63,64].

**Figure 5:**
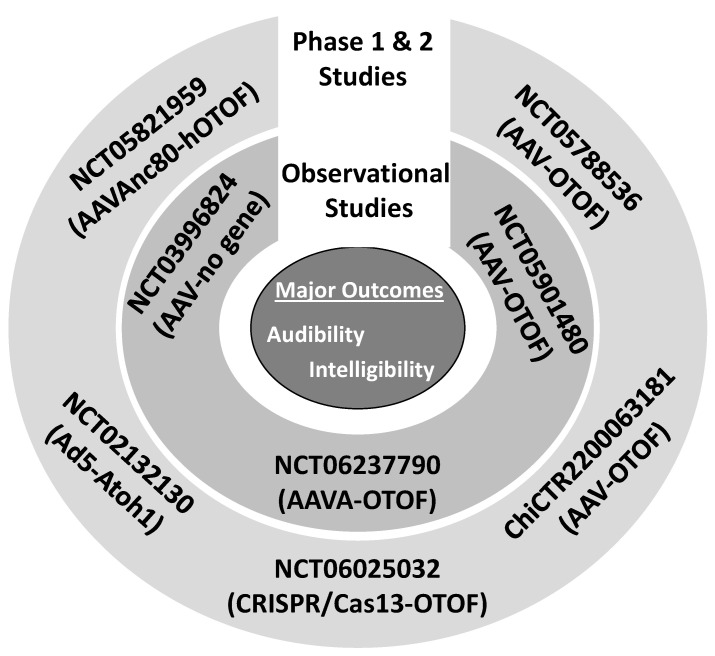
Current gene therapy trials registered in Clinicaltrials.gov (USA) and China Clinical Trials Registry (China). The study number is provided along with the vectors (AAV: adeno-associated virus and Ad: adenovirus), RNA editor (CRISPR/Cas13) and genes of interest (*OTOF* and *Atoh1*). Phase 1 and 2 studies evaluate the safety and efficacy of a given treatment. Observational studies are studies where new data are collected, or existing data are evaluated. The major outcomes for gene therapy studies are complex audiologic phenotypes such as audibility (thresholds, threshold shifts, etc.) and intelligibility (speech perception, comprehension, etc.).

Figure 5 reveals that, currently, there are several registered gene therapy trials. This means that the trend in Figure 4 is expected to continue, which means that clinicians and patients will continue to be inculcated with gene therapy claims. Table 3 provides a rubric for scientists, clinicians, and patients to appraise the strength-of-the-claim made within a given gene therapy study or a given media report. The table lists 10 qualities that a given study or media report must satisfy for the claim to be considered *fair* and if a given study or media report fail to satisfy all 10 qualities then the claim is at best, *mediocre*. Therefore, *fair* is the highest strength that can be awarded to a given claim because appraising evidence claims involves much more than the 10 qualities outlines in Table 3, and thus beyond the time and training of the average clinician or patient. Table 3 provides a simple means (minimum criteria) by which clinicians and their patients can easily appraise the strength of a given claim. The theoretical foundations of Table 3 evaluation framework are grounded in the therapeutic intervention literature (please see the following citations: [73,74,75,76]). News reports tend to be overly biased about gene therapy success. If a given news report fails to address the 10 qualities, then one should question whether the report aims to correctly educate or indoctrinate.

Table 4 deploys a case study to show how the 10 qualities from Table 3 can be directly applied to a real research study. This case study involves one of the most important human trials on gene therapy. The trial was published in one of the most prestigious medical journals (*The Lancet*) and generated an avalanche of media attention and public interest, far beyond what would be expected for research on hearing or hearing loss. The primary claim made in the study is that AAV-*OTOF* gene therapy restored hearing in congenitally deaf children. Unfortunately, the strength-of-the-claim was at best *poor*. The study failed to determine whether the observed hearing recovery after gene therapy would have occurred anyway in the absence of the therapy. For instance, the poor design of the study compels the reader to question whether the five children who showed recovery after the therapy were already poised to show recovery even if the researchers had done nothing or if they had administered tap-water instead of the therapy. This is important because up to 32% of children with congenital hearing loss (including FHL) will exhibit hearing recovery, where moderate to profound hearing loss can progress to normal to moderate hearing loss without biomedical interventions [77,78]. These and other important issues were not considered in this important study, yet the news headlines proclaimed success.

It is meaningful to acknowledge genuine progress in gene therapy because there are many. Among the most prominent is the bacterial/archaea clustered regularly interspaced short palindromic repeats (CRISPR) and CRISPR-associated protein 9 (CRISPR-Cas9) technologies. Unlike routine gene therapy where a single gene with its associated variant is replaced with a normal copy, CRISPR-Cas9 simply edits (akin to correcting a spelling error) the variant within the existing gene. This technology has yielded some success in both pre-clinical research and recent human trials on hemoglobinopathies (e.g., sickle cell and β-thalassemia diseases). Other genuine progress has centered around RNA-, base-, and prime-editing technologies. Unlike CRISPR-Cas9, these newer technologies precisely edit variants within existing genes or RNA without creating deleterious double-strand breaks (iatrogenic DNA damage) or the cumbersome use of donor DNA templates [79]. Even more remarkable is the recent development of drive-and-process (DAP) array architectures for multiplex base-editing and multiplex prime-editing in human cells [80]. Rather than editing single genes or a single variant within a gene, DAP simultaneous edit multiple disease-relevant genomic loci. This is particularly important because patients with familial diseases (including FHL) often exhibit multiple variants across multiple genes. The ability to edit all gene variants at once would be a significant breakthrough in gene therapy. However, the molecular biology definition of a gene is an open-reading-frame circumscribed by an initiation codon (AUG, CUG, GUG, AUA, etc.) and a termination codon (UAA, UAG, UGA, etc.). Ever since the sequencing of the *φX174* bacteriophage genome in the 1970s, we have known about nested genes. Nesting or overlapping genes are whole-genes or parts of genes (3′ or 5′) that reside within other genes, and such nested genes can be found within every human chromosome. This means that editing a single nucleotide within a target gene may result in consequences that are impossible to predict (a simple edit in one gene may end up affecting the function of several other unintended genes). The issue of nested genes is generally overlooked but represents a serious threat to widespread acceptance and use of all gene editing technologies.

RNA technologies appear to be a solution to this problem, and their epidemiological successes were evidenced by their world-wide use during the SARS-CoV-2 pandemic. Unlike base- and prime-editing which are permanent and yield unpredictable outcomes due to nested genes, RNA-editing is reversible and transient. Furthermore, CRISPR-Cas9, base-, and prime-editors all introduces foreign (e.g., bacterial) elements that are immunogenic, while current RNA-editing technologies do not [81]. However, proponents of RNA-editing technologies are ignoring several fundamental caveats. One particulate relates to the fact that RNA and changes to RNA (e.g., RNA-editing) can never consistently determine the function of proteins (the whole-point of routine gene therapy, CRISPR-Cas9-editing, base-editing, prime-editing, and RNA-editing is to control the function of proteins to affect disease outcomes). This is because proteins must adopt tertiary and quaternary three-dimensional conformations to execute functions within cells/tissues and these conformations are not reliably predicted from a protein’s own amino acid sequence, thus, it is fanciful to believe that the more distant nucleotide sequences within genes and RNA can drive protein functions (e.g., catalysis). Indeed, functional protein conformations are driven by nano-, micro- and macro-environments, as well as cognate substrates, thermodynamics, interactors, differences between individual cells, intracellular localization, and a large variety of other cell and molecular variables. This means that all gene therapy technologies (whether gene or RNA based) will never consistently achieve their desired therapeutic outcomes (see Table 5).

## 4. Conclusions

Imagine a company-sponsored study published in one of the world’s highest impact academic journals where the researchers claimed that toothpaste can recover hearing in congenitally deaf children. The researchers did not control for the placebo effect, did not implement a control group or control condition, cherry-picked the participants, failed to reduce bias by ignoring double-blinding and blinded data analysis, failed to show any systematic relationship between the toothpaste dose and hearing recovery, failed to show that the magnitude of the hearing recovery was in fact meaningful, and incidentally the therapeutic toothpaste was administered with two other drugs that are known to recover hearing, and by-the-way, the company was involved in study design, data analysis, data interpretation and publishing the manuscript. *Would you really believe that toothpaste can restore hearing in deaf children*? *Then why do we lower our standards and are so quick to believe similar gene therapy claims where the corresponding study was staged in the exact same manner as outlined for the toothpaste study*? The answer can be traced back to the Human Genome Project (HGP: 1990 to 2003) which involved six countries (USA, UK, France, Germany, Japan, and China) and a gaggle of scientists from different disciplines. This effort was marketed as the most important scientific project in the last 100 years. The objective was to sequence (read the genes) the entire human genome. Nobel Prize laureate James Watson proclaimed, “*Since we can now produce good genetic maps that allow us to locate culprit chromosomes and then actually find the genes for diseases, genetics should be a very high priority on the agenda of NIH research… We have to convince our fellow citizens somehow that there will be more advantages to knowing the human genome than to not knowing it*” [82]. Indeed, presidents, prime ministers, politicians, educators, the NIH, scientists, clinicians, influencers, company stakeholders, media outlets, reporters, and many more interested parties were exceedingly successful at conveying genetic deterministic claims (genes cause everything) to the public. Some of the claims regarding the god-like power and benefit of sequencing human genes were to, “*understanding how life works and how disease occurs*”, and “*understand the human body fully*”. According to Francis Collins, director of the NIH, “*Your DNA helix, your language of life, can also be your textbook of medicine. Learn to read it. Learn to celebrate it. It could save your life*” [83]. To date, none of these claims have proven to be true; however, such claims were needed to justify the 13-year effort and over USD 3 billion in expenses. Of course, we already knew that sequencing the genome would not be the *deus ex machina* (savior) of human health and well-being, because *Caenorhabditis elegans’* entire genome was already sequenced and even today we are no closer to understanding the phenotypes of this simple worm than we were before its genome was sequenced. While we have been distracted by research efforts focused on genetic cause and effect, the real biologic determinants of health and disease have been overlooked [8]. It is unlikely that we will change course and reassess because the current *status quo* is too seductive, too scientifically lucrative, and too commercially beneficial. Indeed, more gene therapy studies are coming online which means more hyperbolic therapeutic claims will need to be made. At *prima facie*, one may ask, *so what’s the harm with a few hopeful claims*? Unfortunately, those armed will be patients, their clinicians, and the profession. Patients are repeatedly traumatized by a cycle of false hope (Figure 4) and some patients are enticed into decisions that are at best uninformed. Clinicians will ultimately have to deal with the fallout (dashed hopes, disappointments, loss of trust, litigation, etc.). From the perspective of other biomedical disciplines looking-in, they will simply be amused by the low standards of our profession while devaluing our efforts to the level of pseudoscience. This will ultimately have consequences for the future of the profession in terms of future government policies, future funding, and future growth.

In regard to future directions, it is first important to acknowledge that any form of human hearing loss is a multivariate phenomenon, where multiple variables conspire to produce the hearing loss and the weights of individuals variables are different between individual patients, between individual cochlea (e.g., right vs. left), and between individuals tissues within a given cochlea (e.g., sensory vs. non-sensory epithelia) [8]. This is the reason why two individuals can be exposed to the same exposure at the same time, yet one develops hearing loss while the other does not. Or, why two individuals harboring the same so-called “*deafness gene*” may exhibit completely different clinical presentations (e.g., one person has no hearing loss while the other is deaf). Or, why a given patient may harbor a variant in a so-called “*hearing loss gene*”, yet one ear exhibits normal cochlear morphology and normal hearing while the opposite ear exhibits cochlear malformation and profound hearing loss (see also the audiologic characteristics described above in the Introduction). The current resolute focus on genes as the sole causal variable of FHL or any other type of human hearing loss precludes serious explorations of other intervening variables. Our modern practice of interpreting genetic associations and genetic correlations as genetic causation combined with the single-minded focus on using a single gene (gene therapy) to cure hearing loss is less than convincing. This does not mean that gene therapy is irrelevant. Rather, it suggests that gene therapy may be best positioned as a complement to existing (hearing aids, cochlear, and brainstem implants, etc.) and future (DNA repair therapies, nutraceuticals, etc.) multifactorial therapeutic regimens. Indeed, this notion is hardly without precedent, given that some gene-centric biomedical fields eventually learned this lesson (although reluctantly).

Another important future direction relates to responsible and evidence-based clinical integration of new therapies. For the sake of our patients, clinicians must deploy critical thinking and the highest standards when considering any new therapy. For example, let us re-consider the current movement around the *OTOF* gene as a cure for congenital hearing loss. Variants in the *OTOF* gene are associated with a special auditory condition called auditory neuropathy spectrum disorder (ANSD). ANSD is a condition where cochlear and other ear structures appear to be normal yet retrocochlear structures (synapse, nerve, and central neural networks) may exhibit permanent morphological alterations such as dysplasia, degeneration, and complete absence. For instance, at least 64% of patients diagnosed with ANSD may exhibit permanent morphological alterations to retrocochlear structures which is likely to be an underestimate due to the limits of routinely used methodologies [84,85]. In the current *OTOF* gene therapy trials [6,7,86], the patients are born with severe to profound ANSD (presumably due to permanent degeneration of retrocochlear structures), *so how is it possible for a single OTOF gene to restore the degenerated or missing structures to improve hearing within weeks after the therapy*? Another unresolved question is, *what is the exact mechanism of action for the gene therapy within each patient given the highly heterogeneous* (*unilateral* vs. *bilateral, multiple sites of lesion, multiple genomic loci, multiple etiologies,* etc.) *nature of ANSD*? Given that individual clinicians are ultimately responsible for clinical integration of new therapies, each clinician must, in the future, be exceedingly critical of the available evidence.

## Figures and Tables

**Table 1:** Standard nomenclature for human sequence variations.

General Variant Descriptions (applies to g, c, n, and r; only g used in the examples below):	
g.23A>C	A to C change (substitution) at nucleotide 23 in genomic DNA sequence.
g.23_33del	The 23rd to 33rd nucleotide sequences have been deleted.
g.23_33inv	The 23rd to 33rd nucleotide sequences have been replaced with nucleotides (2 minimum) that are the reverse-complement of the normal sequence (e.g., normal = CTCGA and reverse-complement = TCGAG).
g.23_33dup	The 23rd to 33rd nucleotide sequences are duplicates of previous nucleotides (3′ of original sequence).
g.23_33insGCAT	The nucleotides GCAT have been abnormally inserted between the 23rd and 33rd nucleotide sequences immediately before the normal sequence (5′ of the original sequence).
g.23_33con53_63	The nucleotides between the 23rd and 33rd nucleotide sequences have been deleted and replaced (converted) with a copy of nucleotide sequences between the 53rd and 63rd nucleotide sequences (deletion–insertion).
g.23_33delinsGC	Two (must be more than one) normal nucleotides have been deleted between the 23rd and 33rd nucleotides and replaced with GC (insertion).
DNA Sequence Variations (applies to g and c; also see general variant description above):	
g.23A[12]	A repetitive stretch of DNA starting at the 23rd nucleotide with the number of repeats in brackets.
c.[23C>T; 63G>C]	Two variants on one molecule (cis).
c.[23C>T]; [63G>C]	Two variants on two different molecules (trans).
g.[23C>T(;) 63G>C]	Two variants with unknown phase.
g.23A=	Variant screen performed but no change detected.
RNA Sequence Variations (applies to r; also see general variant description above):	
r.23a>u	The 23rd nucleotide sequence within RNA is normally an “a” but it has been changed to a “u”.
r.[23a>u, 63_73del]	Two RNA variants (substitution and deletion) resulted from a single DNA change.
Protein Sequence Variations:	
p.T23*	A termination codon (Ter or *) exist at the 23rd amino acid (also known as. nonsense change).
p.A23S	The 23rd amino acid (A) is substituted with S (also known as missense change).
p.(A23S)	The protein change is predicted (no empirical proof).
p.A23_S33del	Deletion of amino acids 23 to 33.
p.A23_S33dup	Duplication of amino acids 23 to 33.
p.A23_S24insWT	Insertion of the W and T amino acids at the 23 and 24 place.
p.A23_S24delinsWT	Deletion-insertion (indel).
p.(A23fs)	Frame shift (fs).
p.A23SextW-12	Extension.
p.A23[22]	Repetitive amino acid stretch, 22 repeats starting with the 23 amino acids.
p.[A23*;S33W]	Two variants on one molecule (cis).
p.[A23*];[S33W]	Two variants on two different molecules (trans).
p.[A23*(;)S33W]	Two variants with unknown phase.
p.(T23=)	No predicted consequence at protein level.

DNA produces RNAs which then produces proteins. Both DNA and RNA are composed of smaller substances called nucleotides while proteins are composed of smaller substances called amino acids. Both nucleotides (abbreviated G, C, A, T and U) and amino acids (abbreviated T, R, W, C, etc.) are arranged in sequence (one-by-one). A mutation in the DNA sequence is expected to be transmitted to RNA and protein sequences. Abbreviations: g = 1st nucleotide of genomic reference sequence; c = 1st nucleotide of start codon within the coding DNA reference sequence; n = 1st nucleotide of the noncoding DNA reference sequence; m = 1st nucleotide of the mitochondria DNA reference sequence; r = 1st nucleotide of the start codon within coding or noncoding RNA reference sequence; p = 1st amino acid of reference protein sequence; > = substitution; del = deletion; inv = inversion; dup = duplication; ins = insertion; con = conversion; delins/indel = deletion-insertion. A reference sequence is the normal sequence. A coding sequence has a translation initiation codon (ATG) and a termination codon (TGA, TAA, or TAG). Remarks: DNA (or RNA) sequence variations can never reliably predict the function of the resulting protein. For example, nucleotide substitutions (missense) can produce both loss-of-function and gain-of-function (including dominant negative) proteins. Each determination of clinically significant variants must be empirically determined in biologic assays conducted on each patient.

**Table 2:** Useful resources.

ASHA Continuing Education Course: “Gene Therapy: Current Promises and Future Challenges”. https://apps.asha.org/eweb/OLSDynamicPage.aspx?Webcode=olsdetails&title=Gene+Therapy%3A+Current+Promises+and+Future+Challenges (accessed on 20 March 2024) Note: audiovisual course that provides a modern and balanced perspective on gene therapy for students, clinicians, and educators.
ASHA Voices: “The Limits of Our Genes”. https://leader.pubs.asha.org/do/10.1044/2022-0324-podcast-gene-therapy-three/full/ (accessed on 20 March 2024). Note: Podcast that presents how genes work and what can be realistically expected from gene therapy. Appropriate for clinicians, their patients, students, and the public.
“Animal Models for Understanding Hearing Loss”. In Health and Hearing, pp. 44–78. 2024. https://doi.org/10.1142/9789811265006_0002 [8] Note: a modern treatise on the systems biology of human hearing loss, models of hearing loss (including genetic hearing loss), therapies for hearing loss and how to appraise hearing loss research. Appropriate for students, clinicians, researchers, and educators.
Hereditary Hearing Loss Homepage. https://hereditaryhearingloss.org/ (accessed on 20 March 2024) Note: website that provides an up-to-date overview of the genetics of hereditary hearing impairment for researchers and clinicians working in the field.
Online Mendelian Inheritance in Man^®^ https://omim.org/ (accessed on 20 March 2024) Note: a comprehensive database of all known genetic disorders (including hearing loss). Both genotype and clinical phenotype is provided for each disorder. Appropriate for students, patients, concerned parents, clinicians, scientists, and educators.
HGVS Nomenclature. https://hgvs-nomenclature.org/stable/ (accessed on 20 March 2024) Note: the HGVS Nomenclature curates an Internationally Recognized standard for the description of DNA, RNA, and protein sequence variants that is used to convey variants in clinical reports and to share variants in publications and databases. Appropriate for clinicians, researchers, educators, authors, and students.
Deafness Variation Database. https://deafnessvariationdatabase.org/ (accessed on 20 March 2024) Note: a comprehensive guide to genetic variation in genes known to be associated with deafness. Appropriate for genetic researchers, students, and clinicians.
RefSeq: NCBI Reference Sequence Database. https://www.ncbi.nlm.nih.gov/refseq/ (accessed on 20 March 2024) Note: a comprehensive, integrated, non-redundant, well-annotated set of reference sequences including genomic, transcript, and protein. It is appropriate for students, researchers, authors, and educators.
The Human Protein Atlas. https://www.proteinatlas.org/ (accessed on 20 March 2024) Note: a comprehensive map of all human proteins in cells, tissues, and organs. It is appropriate for students, researchers, authors, and educators.

**Table 3:** Rubric for clinicians and patients to appraise the strength-of-the-claim made within any given gene therapy study or any given media report on gene therapy.

Study or News Report:		
Claim:		
Quality	Description	Quality Score (1 or 0)
Human Participants	Humans were treated with the therapy.	1
Randomization	The required number of human participants were selected randomly and then randomly assigned to groups, such as control groups (groups of participants who do not receive the therapy) and treated groups (groups of participants who receive the therapy). Therefore, each participant had an equal chance of being selected for the study and assigned to any group.	1
Double-blind	Neither the researchers nor the participants were aware of the type of treatment administered to the participants. Therefore, the researchers are not able to bias the results by selecting only patients who have certain attributes (such as conductive hearing loss, fluctuating hearing, etc.) that would interfere with the true effect of the therapy.	1
Control	A group or condition that served as a natural standard by not being exposed to the therapy, surgery, injections, drugs, and/or any experimental procedures. In general, a control group does not receive the therapy.	1
Placebo	A group or condition that served as an artificial standard by not being exposed to the real therapy but exposed to a fake therapy that is almost indistinguishable from the real therapy. Also, the placebo group or condition must be exposed to the same procedures (surgery, injections, drugs, etc.) as that of the real treatment group/condition. Therefore, the placebo is a condition that has no therapeutic value.	1
Dose–response	The therapeutic effect (e.g., restoration of hearing) systematically increases as the therapy increases. This means that as the dose or frequency of the treatment increases then the hearing loss should simultaneously decrease.	1
Meaningful Effect	The therapeutic effect (e.g., restoration of hearing) is large enough to be significant to the communication abilities of the participants in real-world social situations and to mandate changes in clinical management. This is important because changes that are statistically significant can be clinically useless. Therefore, patient outcomes from a given therapy must be meaningful to the patients and directly impact their day-to-day activities.	1
Safety	The therapy was safe for 100% of the participants, monitored over years. No gene therapy can be considered safe without several years of monitoring for cancer development, immunotoxicity and many more side-effects.	1
Confounds	The absence of other variables (drugs, surgery, etc.) that could drive a meaningful effect, instead of (or in addition to) the gene therapy. This means that the researchers were not able to bias the results of the clinical trial by including, drugs, procedures, or other therapies that would impact the hearing loss and lead to the false assumption that the therapy restored hearing.	1
Blinded Analyses	The analysis of the data was conducted by research personnel who were unaware of which data belonged to which group, condition, and placebo. This means that the researchers could not bias the data analysis to find positive effects of the therapy when there is no positive effect.	1
Strength-of-the-claim score (underline one) → Fair Mediocre Poor		
To calculate strength-of-the-claim: (1st) Count the number of ones, then divide by 10, then multiply 100 = percent (%). (2nd) A strength-of-the-claim score of 100% means fair; 99–71% means mediocre; and ≤70 means poor.		

**Table 4:** Case study.

**Study or News Report:** Lancet 2024, S0140-6736(23)02874-X. https://doi.org/10.1016/S0140-6736(23)02874-X [6].		
**Claim:** AAV1-*hOTOF* gene therapy recovered hearing in deaf children.		
**Quality**	**Description**	**Quality Score** **(1 or 0)**
Human Participants	YES	1
Randomization	NO	0
Double-blind	NO	0
Control	NO	0
Placebo	NO	0
Dose–response	NO	0
Meaningful Effect	NO	0
Safety	NO	0
Confounds	NO	0
Blinded Analyses	NO	0
Strength-of-the-claim score (underline one) → Fair Mediocre Poor		
To calculate strength-of-the-claim: (1st) Count the number of ones, then divide by 10, then multiply 100 = percent (%). (2nd) A strength-of-the-claim score of 100% means fair; 99–71% means mediocre; and ≤70 means poor.		

**Table 5:** Key advantages and limitations of each gene therapy approach.

Gene Therapies	Advantages	Disadvantages	Remarks
Routine gene therapy	Seeks to match pathogenic effects (amorphic, hypomorphic, hypermorphic, neomorphic, antimorphic, etc.) from the gene variant to gene therapy strategies (replacement, addition, suppression, editing, etc.) to achieve precision/personalized medicine.	Pathogenic effects that result from pathogenic variants are context dependent, such that the same variant is associated with different pathogenic effects (even normal effects) in different patients/organs/tissues/cells. Therefore, the pathogenic effect from each pathogenic variant must be verified within the target cells/tissues for each patient before selecting a gene therapy strategy, yet this is rarely if ever done and might be impossible in some situations.	All gene therapy technologies (whether gene or RNA based) will never consistently achieve their desired therapeutic outcomes because genes and RNA cannot faithfully control the functions of proteins (malfunctioning proteins are believed to cause genetic diseases so gene therapies seek to correct the proteins by correcting the genes or RNA). This is because a protein’s function is dependent on the environmental context in which the protein is staged. For example, a given protein (p53, ATM, XPC, RPA, XPA, XPF, XPG, etc.) can contribute to both cell death and cell survival depending on how it is post-translationally modified, whether it is localized to the nucleus vs. cytoplasm, the spatio-temporal specifics of its pulsing dynamics, what other proteins it interacts with, etc. Given the unique nature of gene therapies and the circumstances around patient treatment with such therapies, it is well-known that the Placebo Effect (improvement in a patient’s condition from a fake treatment) is particularly prominent in all successful gene therapy clinical trials yet gene-centric scientists, clinicians, and the media choose to overlook or not quantify the magnitude of the placebo effect.
CRISPR-Cas9	Deliberately creates DNA damage within a patient’s genome to make permanent corrections to the patient’s genes.	Falsely assume that the patient’s DNA repair pathways will: (1) correctly repair the CRISPR-Cas9 induced DNA damage, (2) all target cells have the same DNA repair capacity/efficiency, (3) the consequences of failed DNA repair will not result in cancers, toxic immune responses and increased susceptibilities to future/other types of diseases. Yet, adjuvant DNA repair therapies are never used.	
RNA-, Base- and prime-editing	Uses engineered or natural enzymes to facilitate the editing of DNA or RNA.	All engineered and natural enzymes are vulnerable to Star-Activity (the enzyme doing things that we cannot predict) which can occur due to a slight change in temperature, pH, salt/metal concentration, the unit number of enzymes, and over 1000 additional variables. This means that any given enzymes will cut or make changes in areas where it is deleterious to do so and will perform differently in different cells/tissues/organs/patients.	

Definitions: Pathogenic effects refer to protein dysfunctions which can be amorphic (complete loss of protein function), hypomorphic (incomplete loss of protein function), hypermorphic (abnormal increase in protein function), neomorphic (protein adopts a completely new function), or antimorphic (also known as dominant–negative, the protein attacks/inhibits other proteins including normal copies of itself). Gene therapy strategies can be replacement (treatment with a normal copy of the bad gene), addition (treatment with a different gene to compensate for the damaged gene), suppression (treatment to reduce the expression of the damaged gene), or editing (treatment to change the DNA or RNA of the damaged gene).
